# 15 Years MR-encephalography

**DOI:** 10.1007/s10334-020-00891-z

**Published:** 2020-10-20

**Authors:** Juergen Hennig, Vesa Kiviniemi, Bruno Riemenschneider, Antonia Barghoorn, Burak Akin, Fei Wang, Pierre LeVan

**Affiliations:** 1 Department of Radiology, Medical Physics, Faculty of Medicine, Medical Center University of Freiburg, University of Freiburg, Freiburg, Germany; 2 Center for Basics in NeuroModulation (NeuroModulBasics), Faculty of Medicine, University of Freiburg, Freiburg, Germany; 3 Oulu Functional NeuroImaging Group, Research Unit of Medical Imaging, Physics and Technology, University of Oulu, Oulu, Finland; 4 Department of Radiology, Center for Biomedical Imaging, New York University Grossman School of Medicine, New York, NY USA; 5 Departments of Radiology and Paediatrics, Hotchkiss Brain Institute and Alberta Children’s Hospital Research Institute, University of Calgary, Calgary, AB Canada

**Keywords:** Magnetic resonance imaging, Functional magnetic resonance imaging

## Abstract

**Objective:**

This review article gives an account of the development of the MR-encephalography (MREG) method, which started as a mere ‘Gedankenexperiment’ in 2005 and gradually developed into a method for ultrafast measurement of physiological activities in the brain. After going through different approaches covering k-space with radial, rosette, and concentric shell trajectories we have settled on a stack-of-spiral trajectory, which allows full brain coverage with (nominal) 3 mm isotropic resolution in 100 ms. The very high acceleration factor is facilitated by the near-isotropic k-space coverage, which allows high acceleration in all three spatial dimensions.

**Methods:**

The methodological section covers the basic sequence design as well as recent advances in image reconstruction including the targeted reconstruction, which allows real-time feedback applications, and—most recently—the time-domain principal component reconstruction (tPCR), which applies a principal component analysis of the acquired time domain data as a sparsifying transformation to improve reconstruction speed as well as quality.

**Applications:**

Although the BOLD-response is rather slow, the high speed acquisition of MREG allows separation of BOLD-effects from cardiac and breathing related pulsatility. The increased sensitivity enables direct detection of the dynamic variability of resting state networks as well as localization of single interictal events in epilepsy patients. A separate and highly intriguing application is aimed at the investigation of the glymphatic system by assessment of the spatiotemporal patterns of cardiac and breathing related pulsatility.

**Discussion:**

MREG has been developed to push the speed limits of fMRI. Compared to multiband-EPI this allows considerably faster acquisition at the cost of reduced image quality and spatial resolution.

## Introduction

The following gives a narrative account of the development of the method over the years written by J. Hennig.

**Fig. 1:**
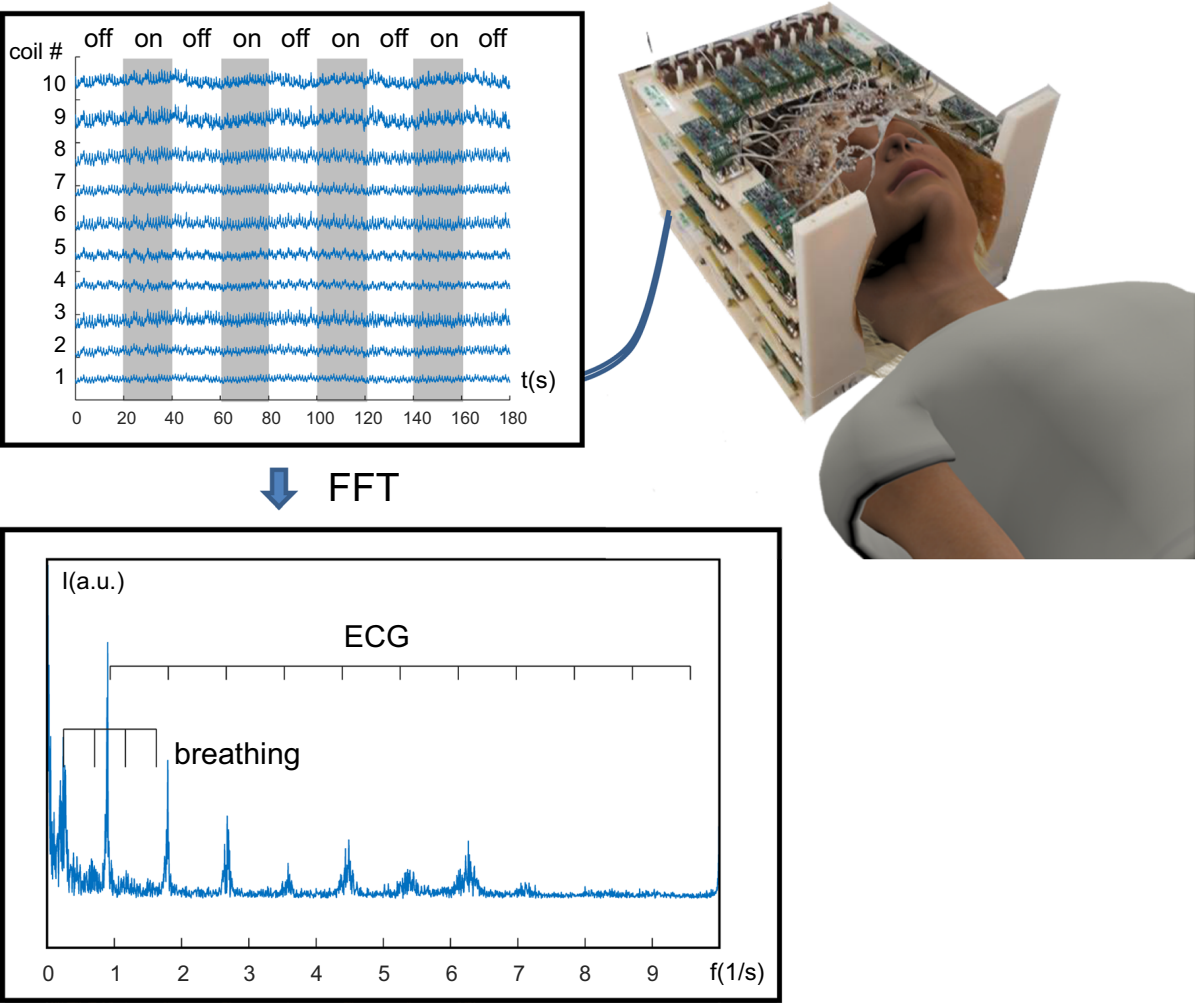
Basic principle of OVOC-experiment. The signal of each coil is separately measured and recorded and can be displayed as multi-channel signal display just as in EEG (top left). Fourier transformation of the signal time course reveals distinct peaks attributed to breathing and ECG-related signal pulsatility. Due to its very ‘spiky’ nature, especially ECG shows pronounced intensity at higher harmonics. Only the spectrum from channel 10 is shown

The idea to acquire images based on the sensitivity of coil profiles alone without any gradients came up during the preparation of the Mansfield lecture at ISMRM 2005 in the Gleason Theater in Miami. My train of thoughts for the ultimate speed limit in MR was as follows: What does it take to encode an image? Gradients. Why is MR imaging still so painstakingly slow? Because of gradients. So how could we get beyond all speed limits for fast imaging? By avoiding gradients. Therefore, where do we get spatial information from? Put like that the answer was pretty obvious. Around that time multi-coil arrays with multiple individual coil elements became available, so why not associate each voxel to one coil element in such an OVOC (one voxel one coil)-experiment [1]? The spatial resolution of such an experiment is of course rather poor and given by the sensitive volume of each coil element, but the temporal resolution suddenly becomes close to unlimited. The OVOC-principle can be applied to any part of the body, but we have more or less exclusively focused on brain applications. Applications for ultrafast BOLD-fMRI as well as measurements of breathing- and ECG-related pulsatility have been the main areas of application so far. Applied to the brain this measurement principle closely resembles the principles of Electroencephalography (EEG), therefore, I named the new method MR-Encephalography (MREG). The basic principle is the same: measure signals within the sensitive range of a detector array. In EEG the array consists of multiple electrodes measuring the voltage induced by postsynaptic currents at the surface of the head, whereas MREG measures the MR-signal under each element of a multichannel coil (Fig. 1).

This way one can acquire MR-signals free from the speed limitations of gradient switching. The measured signals can be presented as signal traces of the individual channels (= coils) just as in EEG. Frequency analysis of the measured signals shows pronounced peaks corresponding to breathing as well as ECG-related signals. Especially the latter shows higher harmonics up to ±5 Hz due to the very spiky nature of ECG–related signal pulsatility. Signal peaks at higher harmonics are spread out more and more due to imperfection in the periodicity of the ECG-pulse.

**Fig. 2:**
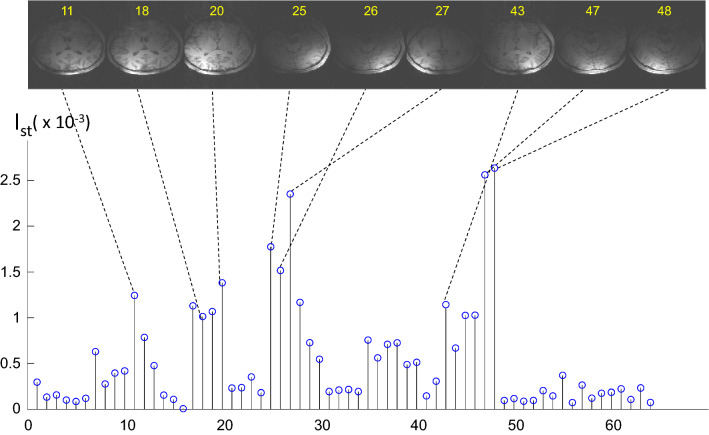
Relative signal intensity I_st_of the spectral peak at the stimulus frequency compared to baseline in a visual stimulation experiment in the individual coil elements of the 64-channel coils used. Sample images of coils with high intensity show that the respective coil elements cover the visual cortex. The relative change is rather small, since the activated voxels represent just a small fraction of the total signal in each coil

Figure 2 shows the result of such an experiment with visual stimulation by a flickering checkerboard. Frequency analysis of the measured signal traces at the stimulus frequency shows increased signal amplitude in coil elements covering the visual cortex.

**Fig. 3:**
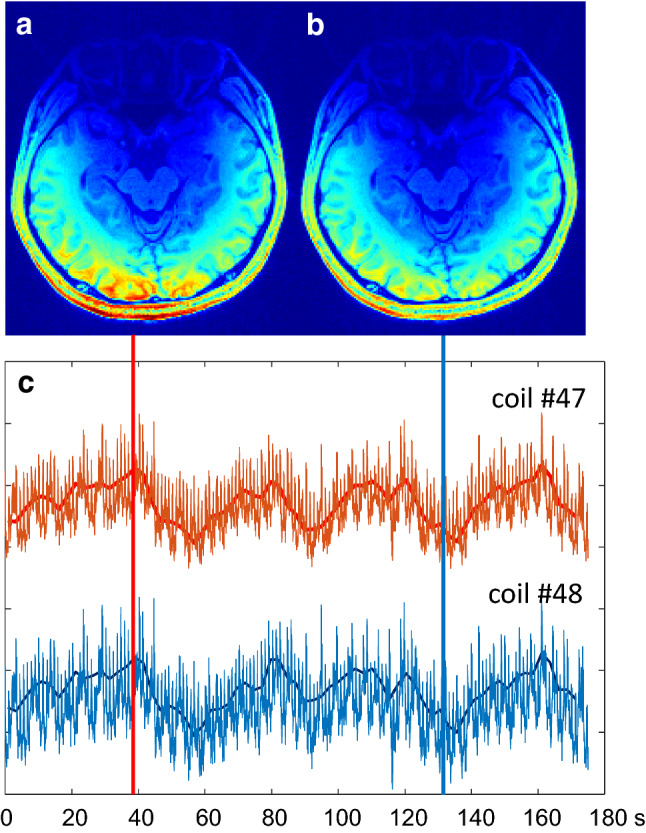
Activation ‘images’ at the top of the BOLD response (**a**) and during rest (**b**). Images are generated by sum-of-squares combination of reference images of all individual coil elements weighted with the actual signal intensity in each coil element. **c** shows individual signal time courses in two coils. Signals have been low pass filtered to reduce ECG-dependent flickering (solid lines in **c**). **a** Corresponds to the time indicated by the red vertical line in **c**, **b** to the time indicated by the blue vertical line

Compared to EEG a MREG-measurement has some intrinsic advantages with respect to localization of the signals: In EEG the problem of source localization is a highly underdetermined inverse problem, which produces an infinite number of solutions depending on the chosen boundary conditions. In MREG we can use a conventional MR-image acquired prior to the experiment to set at least some boundary conditions to where the signal is coming from. A rather direct way to turn the signals into images is to multiply independently acquired coil images under the respective coil with the respective actual signal amplitude of the MREG-acquisition and then do a sum-of-squares combination of the individual coil images (Fig. 3). This way one can ultimately reconstruct an image from a single data point. Chuck Mistretta, who has adapted this principle in his HYPR-scheme [2], was the first to point out that this way the signal to noise ratio of the resulting image does not depend on the acquisition time of the time-resolved scan, only on the template image. He also coined the term ‘Hennig limit’ for reconstructing an image from a single data point.

Of course, the spatial information about the location of the dynamic signal variation in such an OVOC-experiment is rather coarse, but with this approach an imaging speed in the megahertz range would be perfectly feasible using a standard analog-to-digital converter (ADC). There are no physiologically meaningful signals at such high speed, so one can invest some of the speed gain into a little bit of spatial encoding. Initially the intention was not to make images in the true sense, but I thought it would be neat to acquire data under, e.g., some ‘depth encoding’ gradient, which encodes for the depth localization of some activation signal under the skull or by a tangential gradient which would give the lateral position with respect to the coil (Fig. 5 in [1]). In this context it would make a lot of sense to have the coordinate system of the gradients oriented locally to the geometry of each coil element rather than globally in the usual *x*, *y*, *z*-directions. This led to the concept of parallel imaging in non-bijective, curvilinear magnetic field gradients [3] which has become a fruitful field of research on its own, culminating in the 84-channel matrix coil designed and built by Maxim Zaitsev [4–6]. It has to be shamefully admitted that up to now I haven’t succeeded in bringing these two concepts together, still too busy optimizing each component on its own. Bringing the concepts of local magnetic fields together with highly parallel coil arrays is a considerable technological challenge to be explored in the future development of the method.

**Fig. 4:**
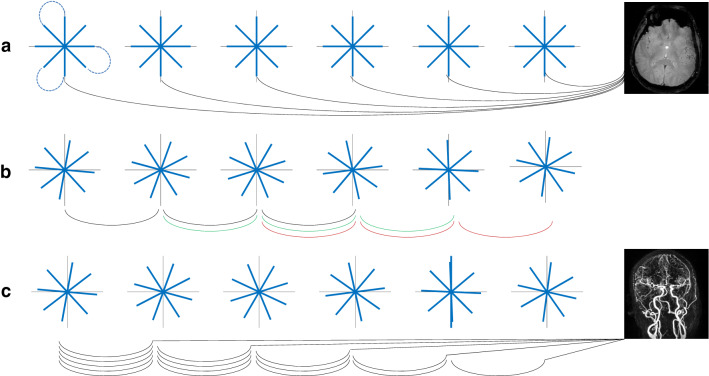
Sampling schemes of COBRA (**a**) compared to VIPR (**b**) and HYPR (**c**). The lines under the trajectories indicate the reconstruction strategy: COBRA uses repetitive sampling of identical radial spokes with reconstruction of each individual timeframe to preserve the temporal fidelity of the scan, VIPR is based on a sophisticated view sharing scheme with a trade-off between temporal and spatial resolution (colored lines indicate combinations of data used for reconstruction of individual time frames), whereas HYPR generates images with high temporal resolution by weighting the final high-resolution image with the low-resolution image of individual time frames. Note that in all sampling schemes acquisition runs continuously, the collection into packages of 4 spokes is for visualization only. The dotted lines in the top left diagram in **a** illustrate how multiple radial spokes are converted into a single shot rosette trajectory

A sum-of-square reconstruction of weighted template images is of course a rather crude way to exploit the information content of the measured signals. It does not consider that the individual coil profiles show considerable overlap, which—together with the coil sensitivities—can be exploited using parallel imaging reconstruction. Data acquired with one or a few projections are highly undersampled and yield very poor image quality when directly subjected to SENSE reconstruction. Miki Lustig had published his seminal paper on compressed sensing in 2007 [7] where he also mentioned in the discussion that this can be combined with parallel imaging, but I didn’t feel sufficiently confident with this new approach to use it for our specific problem. It was not quite clear to me, whether the very scarce but rather regular undersampled acquisition of a few projections would conform to the sparsity constraints which seemed to be essential for compressed sensing. I was lucky to know, however, J. Honerkamp, Professor for Theoretical Physics at Freiburg University, who had supported me a lot in the early days of setting up my group. He has published numerous papers and books on how to approach ill-posed inverse problems [8] using techniques like Tikhonov regularization [9]. So Thimo Hugger (publishing under his birthname Grotz), my first PhD-student working on MREG, implemented the image reconstruction as the regularized solution of an inverse problem to improve the spatial definition of our ‘images’. The result was what we aptly called the COBRA-sequence [10]—a highly undersampled radial acquisition technique using only 2–4 projections per time frame. The sampling scheme of COBRA is very similar to that of VIPR [11] and HYPR [2], all of which use an undersampled radial acquisition scheme (Fig. 4). In COBRA the same few projections are acquired again and again, and the image is reconstructed using regularization using a previously acquired reference image as template. VIPR uses a golden angle approach to radial sampling, where the acquired data can be binned together such that a tradeoff between temporal and spatial resolution can be made during reconstruction. VIPR reconstruction does not require a template image as a constraint during reconstruction. HYPR uses the same acquisition scheme as VIPR but images are reconstructed by incrementally adding multiple projections and using a constrained reconstruction based on the fully sampled dataset which results from all (or a large number of) projections.

Until then gradients had been used to merely improve the spatial localization of the signals and not necessarily to produce images. The COBRA-results were very encouraging, however; quite accurate activation maps could be reconstructed from as few as 3–4 projections (Figs. 7, 8 in [10]). The frequency analysis of the signal time courses clearly showed the quite strong signals from breathing and ECG pulsatility already observed in the OVOC-experiments. The high pulsatility of the ECG-dependent signals shows strong higher harmonics up to 5 Hz. Based on these findings we set our goal to develop spatial encoding schemes which allow 3D-acquisition within a TR of not more than 100 ms which corresponds to a Nyquist limit of ±5 Hz.

VIPR and HYPR are typically applied in 3D-mode. Extending the COBRA-approach to 3D by acquiring one projection per excitation would not be very time efficient especially given the long echo times necessary to achieve BOLD contrast. Thimo Hugger together with Benjamin Zahneisen showed that multiple projections can be acquired in a single shot acquisition by connecting the ends of the single projections of a 3D sampling scheme which leads to a rosette trajectory [12, 13] (Fig. 4).

The resulting isotropic 3D-volumes showed very high sensitivity to detect functional activation. The quality of the underlying images is quite reasonable over most of the brain but shows pronounced signal loss in areas with strong susceptibility effects and field inhomogeneities, which can only be gradually improved using a field map correction. Closer analyses revealed that the main cause of the signal loss lies in the multiple self-crossings of the trajectory in the (nominal) center of k-space. Even small off-resonance effects lead to data inconsistencies with subsequent signal loss and potential artifacts.

As the next step, we, therefore, turned to a non-intersecting concentric shell trajectory [14, 15], which led to a considerable improvement of the point spread function compared to rosettes (Fig. 4 in [15]). The trajectory has an isotropic PSF which is beneficial for brain wide studies as in resting-state fMRI (rsMRI). As shown in Fig. 6 in [15] images still show signal voids in areas of strong field inhomogeneity, but to a much lesser extent compared to rosettes. Since the non-intersecting trajectory avoids data inconsistencies, field map correction works much better.

**Fig. 5:**
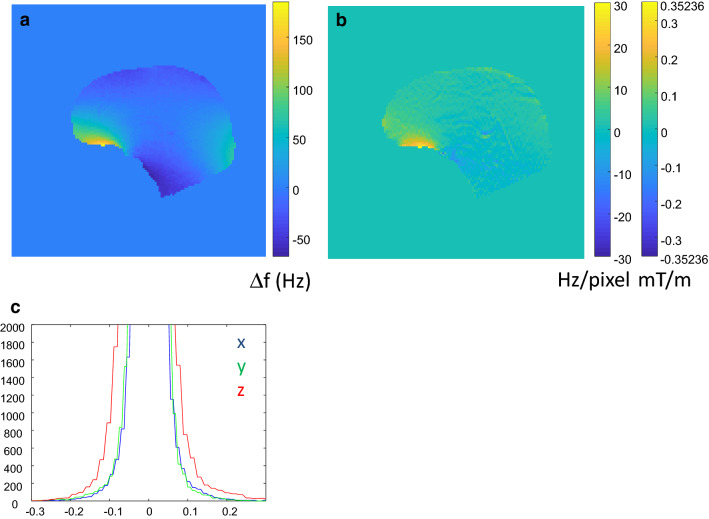
**a** Field map and (**b**) map of susceptibility induced field gradient over the brain. **c** Shows the histogram of the pixel count as a function of the field gradient clearly demonstrating the dominance of susceptibility effects in the *z*-direction

The isotropic trajectory shows isotropic sensitivity to susceptibility induced field gradients. It is known, however, that susceptibility induced field changes are strongest along the main direction of the field (*z*-direction). This follows from the Biot-Savart equation and is also demonstrated experimentally by plotting the histogram of susceptibility induced field gradients in the *x*-, *y*- and *z*-direction (Fig. 5). Based on this consideration Jakob Asslaender came up with the idea to implement a single-shot stack-of-spirals trajectory, which is isotropic in *x*, *y* but shows a more benign off-resonance behavior in the *z*-direction [16]. At first sight it appears to be counterintuitive to use the *z*-direction—where susceptibility gradients are strongest—as the slowest encoding direction. The rationale is given by the monotonous trajectory along z which leads primarily to a susceptibility induced shift and only to a minor degree to some blurring and signal loss. The influence of off-resonance effects and susceptibility gradients is discussed in detail in [16]. Due to the 3D-acquisition inflowing blood is already at least partially saturated. A strong spoiler after the read out trajectory is used to minimize spin history effects from flowing blood. As shown in Fig. 12 no signals from vessels are directly observed, but—predominantly arterial—signals clearly still contribute to the observed signal variation. The observed pulsations are thus likely a combination of spin history effects (especially in CSF regions, where T2 is long) and actual local pulsatility [17]. The resulting sequence has meanwhile—with some modifications—become our workhorse sequence for all MREG-applications described in the following chapters.

**Fig. 6:**
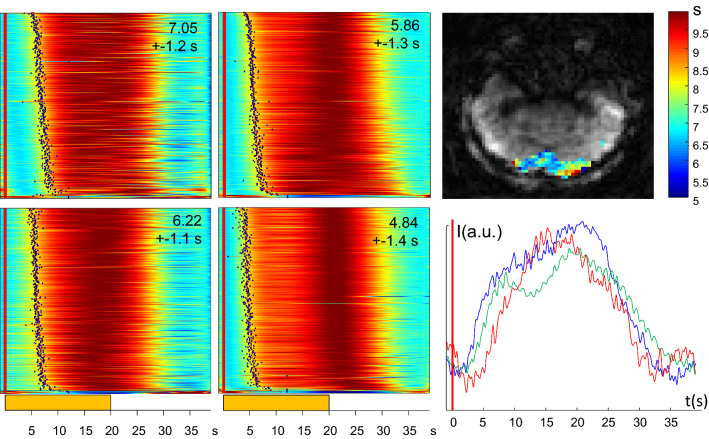
Color-coded image display of four consecutive activation periods of visual stimulation with a flickering checkerboard (20 s on–20 s off). Each frame shows normalized signal amplitudes in activated voxels (thresholded at *t* values > 30). The number in each frame represents the mean and standard deviation of the BOLD arrival time over the activated signal time courses. The BOLD arrival time is measured as the time at which the signal reaches half its maximum in each stimulation period. The yellow bar at the bottom indicates the stimulus-on period, which starts 1 s into each frame (vertical red lines). The BOLD arrival time map representing the mean arrival time over 4 stimulation periods is shown on top right, the color bar represents the BOLD arrival time in seconds. At bottom right three signal time courses at different BOLD arrival times are displayed. Numbers in each frame represent the mean BOLD arrival time and standard deviation for each stimulation period. The mean arrival time over all 4 periods is 5.99 ± 1.25 s

Figure 6 shows an example from mapping the BOLD response in the visual cortex and demonstrates the high sensitivity of the technique and its ability to observe and characterize individual activation time courses. Signal time courses have been sorted according to the mean arrival time over all 4 stimulation periods. Bolus arrival time was calculated using the procedure described in [13]: First PCA is applied on all activated signal time courses. Individual pixel time courses are then modelled by the first *n* PCA components, *n* = 10 was used here. Pixel timecourses have been smoothed and normalized for display. The considerable variability of the BOLD response in different voxels is clearly demonstrated. In the dataset shown, the onset time of the BOLD response shows a regional variation with a general tendency for the BOLD response to arrive earlier in the central part of the visual cortex compared to the peripheral part. The shape of the HRF also shows a considerable variation across the activated region. These results are far from definite and were shown just for illustration of the capacity of the method to detect functional activity of single activations in single subject measurements. BOLD onset times between different pixels are very consistent within each activation period with some distinct variation between activations.

The development of MREG didn’t take place in a vacuum, but was accompanied by various approaches of other groups to drive the speed limits of (f)MRI. At the time of the original conception of the principles of OVOC in 2005, Steve Wright had just published his single echo acquisition [18] which is based on an array of 64 strip-like coils arranged row by row such that gradient encoding for 2D-imaging had to be applied only in the direction of the strips, the 2nd direction was resolved by the coil array alone. Indeed later I learned that the principle of OVOC had been published already in 1988 by Hutchinson [19]—long before the advent of multi-coil arrays and parallel imaging. The paper presented purely theoretical simulations based on expected individual coil profiles placed within each pixel of a MR-image ignoring the practical problem of how to actually place an RF-coil within the tissue. Coupling between coils was ignored but in the discussion a number of quite up-to-date measures were described to minimize coupling between coil elements. It even mentioned that with a proper solution to the coupling problem one might be able to ‘…employ a nest of large overlapping detectors in which case the signal-to-noise ratio might be largely preserved…’. This barely missed the invention of the phased array published by Peter Roemer 2 years later in 1990 [20].

In parallel to my own work Fa-Hsuan Lin worked on what he called Inverse Imaging (InI), the publication of which in fact preceded the first MREG-paper and won him the Rabi-Award in 2006 [21–25]. In contrast to our isotropic approach he used standard parallel imaging with gradient encoding in the *y*–*z*-(sagittal)-plane and pure coil encoding in the *x*-direction, which resulted in a very anisotropic PSF, but still achieved very short acquisition times. A generalized version of InI has been suggested by Boyacioğlu [26].

Cartesian single-shot 3D acquisition—echo volumar imaging or 3D-EPI—has been around a long time [27] and also been used for fMRI [28]. It doesn’t quite reach the temporal performance of MREG, but in combination with controlled aliasing and multi-slab acquisition short repetition times down to 371 ms have been reached [29]. A ‘virtual’ increase in temporal resolution by time shifted acquisition has been demonstrated in [30].

As time went by, simultaneous multi-slice (SMS)-EPI was introduced [31–34], which doesn’t quite match the temporal performance of MREG but yields much better image quality [35] due to the rather benign artifact behavior of the Cartesian k-space trajectory [33, 36–38].

Most recently other groups have been working on ultrafast spiral imaging. A repetition time of 200 ms at 2.8 mm isotropic resolution has been reported using a T-Hex single-shot spiral trajectory [39] and 3.5 ms/slice could be achieved with 2D-spiral imaging using a dedicated gradient coil [40].

## Materials and methods

**Fig. 7:**
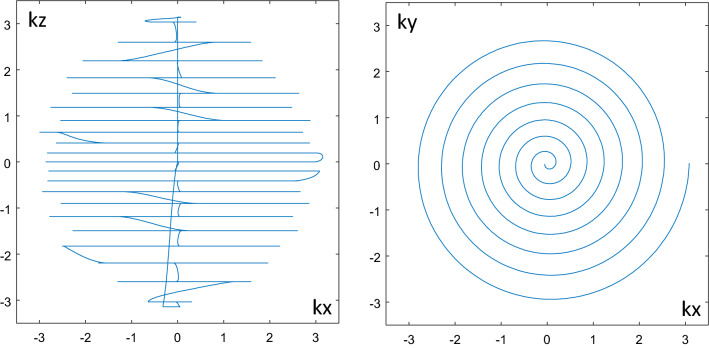
Projections of the actual k-space trajectory onto the *kx*-*kz*-resp. *kx*-*ky*-plane

The k-space trajectory currently used is shown in Fig. 7. It consists of a stack of 21 spirals inscribed within a sphere. The distance between spirals increases with distance to the k-space center, individual spiral elements are sampled with variable density. Details about the construction of the trajectory are given in [16].

In a typical acquisition a total of 14,908 data points are sampled with a bandwidth of 200 kHz leading to a total acquisition time of 74.54 ms. Echo time is 33 ms (k-space origin is reached at the beginning of the central spiral segment). With a TR of 100 ms the flip angle is adjusted to 21° corresponding to the Ernst angle for gray matter.

Data are acquired with a 64-channel headcoil, of which 40 coil elements cover the brain. Based on the reconstructed volume of size 64 × 64 × 64 the undersampling factor is ~ 18 or 2.6 per direction. Direct application of parallel reconstruction using eg non-Cartesian SENSE shows some artifacts due to the high undersampling factor; therefore, a regularized reconstruction is used. Nominal spatial resolution is 3 mm isotropic, but the actual resolution is lower and anisotropic due to the nature of iterative reconstruction of the highly undersampled acquisition. Details are found in [16].

For reference a standard double echo gradient echo sequence is typically performed prior to the MREG-acquisition, which is used to calculate coil sensitivity profiles as well as a 3D fieldmap. Example settings at 3 T are: FOV = 192 mm: TR = 1000 ms, TE1 = 2.3 ms, TE2 = 4.6 ms, α = 50° and 64 slices (slice thickness = 3 mm), which yields a 3D-dataset of identical matrix size as the MREG-data in in 64 s.

Coil sensitivities are calculated by employing the method by Walsh [41] from the same dataset. For fieldmap calculation coil images are combined voxelwise based on the maximum intensity of the absolute values over all coils, the field map is then calculated from the phase difference between the two echoes. Phase unwrapping is performed with PRELUDE [42] provided by the FSL toolbox (https://www.fmrib.ox.ac.uk/fsl).

### Reconstruction

Using the notation from [43] the general signal equation is written as

1 $$s = E m,$$

where *s* represents the measured signal in all coils, *E* is the forward operator representing the measurement process and m is the image to be reconstructed. Equation (1) is solved by regularized iterative reconstruction minimizing the cost function *f*(*m*):

2 $$f\left( m \right) ={\parallel} Em - s{\parallel}^{2} + \lambda_{n}^{n} {\parallel}{\Psi }m{\parallel}_{n}^{n} .$$

Here, ${\Psi }$ indicates some (optional) sparsity transform such as total variation (TV) or wavelet transform, $n$ could be 1 or 2 representing L1- or L2-Norm. Data in most practical applications are reconstructed without additional sparsity transform, i.e., ${\Psi }$ corresponds to the identity matrix.

The reconstruction framework for iterative reconstruction had already been set up for the previous trajectories. A detailed description is found in [12]. The forward operator *E* is implemented as an operator that first multiplies the image with the coil sensitivities and thereafter performs a non-uniform fast Fourier transformation (NUFFT) with min–max interpolation [44]. Off-resonance correction is performed in a segmented approach described by Sutton [45].

Various penalty terms for iterative reconstruction have been explored, most often used is Tikhonov regularization which is based on a linear conjugate gradient (CG) algorithm with L2 norm and L1-norm regularization. The latter uses a non-linear conjugate gradient algorithm, which takes much longer but leads to better image quality by its edge-preserving nature. Transformation in the wavelet domain did not improve performance, most likely due to the non-random sampling trajectory.

Global frequency changes during the acquisition of the time series are corrected by the “dynamic off-resonance in k-space” (DORK) approach suggested by Pfeuffer [46], corrections of field inhomogeneities based on the separately acquire field map.

Image reconstruction takes about 50 s per volume per core without off-resonance correction and up to 10 times longer with off-resonance correction depending on the number of time segmentation steps used. Even on a computer cluster with multiple cores total reconstruction time of the 3–10,000 time frames typically acquired within an experimental session takes several hours or even days if the slower L1-reconstruction is used.

### Real-time implementation of MREG using targeted reconstruction

The application of the ultrafast data acquisition achieved by MREG to real time-feedback applications appears to be very attractive, since it could reduce the long response delays associated with standard acquisitions. However, the long reconstruction times necessary seem to defy such an application. Given this challenge in a collaboration with Rainer Goebel from Maastricht Bruno Riemenschneider came up with the concept of targeted reconstruction [43, 47].

Formally the resulting 3D-image m in Eq. (1) is associated to the measured signal by the pseudoinverse:

3 $$\left( {E*E} \right)^{ - 1} E*s = m.$$

**Fig. 8:**
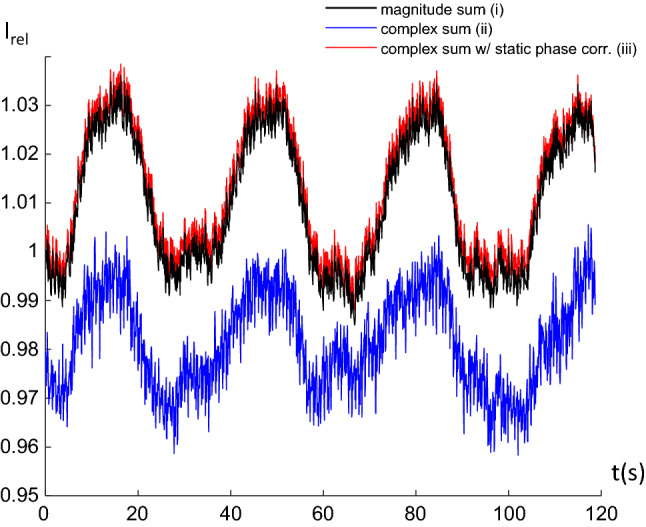
Signal time course over the visual cortex generated as the sum of the magnitude signals of a fully reconstructed dataset (black) compared to the complex sum reconstructed with targeted reconstruction without (blue) and with (red) correction for static phase (same data as in Fig. 5 in ref. [43] were used). Reference signal and complex sum with phase correction are nearly identical, the red line has been slightly shifted in the plot for clarity

If one wants to reconstruct summed data from an arbitrary subvolume of the total image *m*, this can be derived by forming the scalar product of *m* with a vector *V* containing arbitrary voxel weights representing the subvolume. Using basic properties of the scalar product this yields

4 $$\left\langle {m, V} \right\rangle = \left\langle {\left( {E*E} \right)^{ - 1} E*s, V} \right\rangle = \left\langle {s,E \left( {E*E} \right)^{ - 1} V} \right\rangle = \left\langle {s, v} \right\rangle ,$$

where

5 $$v = E\left( {E*E} \right)^{ - 1} V,$$

is a linear combination of lines of the complete reconstruction matrix, weighted by V. This means that the time consuming iterative reconstruction to form the pseudoinverse has to be performed only once, all following signals from the desired subvolume can then be calculated as a scalar product, which is fast. Implementation is pretty straightforward for calculation of single pixel time courses (Fig. 4 in ref. [43]). Reconstruction over larger ROIs with considerable phase variations is more challenging. In fully reconstructed datasets absolute values of pixel intensities are used to form ROI signals. The targeted reconstruction algorithm works inherently on complex data, so any dephasing over a ROI will lead to signal loss. This can be avoided by including a static phase correction which leads to near-perfect retrieval of ROI time courses (Fig. 8).

In the actual implementation it was demonstrated that signals from up to 30 predefined subvolumes can be calculated within the repetition time of 100 ms.

### Time-domain principal component reconstruction (tPCR)

The targeted reconstruction algorithm is very efficient for reconstructing signal time courses within predefined ROIs, but it doesn’t help to accelerate the time consuming framewise reconstruction used for full reconstruction. Fei Wang has developed a method to accelerate the reconstruction by first performing a principal component analysis (PCA) on the raw data, then reconstructing each PCA component followed by recombination of all components to yield the full 3D-time series [48]. At first sight this time-domain principal component reconstruction (tPCR) seems to increase the computational burden by the additional effort to perform PCA on the coil-wise raw data. The rationale why this may still reduce overall reconstruction time was the insight that higher PCA-components contain less and less relevant information and may thus be reconstructed with fewer iterations. In simulation experiments as well as experimentally it could be demonstrated that the number of iterations necessary for reconstruction is higher for the low order PCA-components but decreases quickly for higher order components. For frame-wise reconstruction the number of iterations remains constant as individual timeframes are highly similar to each other. As a result the overall number of iterations necessary for tPCR is considerably reduced.

For a linear reconstruction algorithm the recombined PCA-components should be exactly identical to the result of frame wise reconstruction. Since iterative reconstruction introduces some nonlinearity in the reconstruction process, this is not necessarily the case for tPCR. The results of the simulations shown in [48] indeed demonstrate that tPCR is not only faster but also reduces the reconstruction error. This can be explained by the fact that PCA represents the densest expression of data leading to an overall increase of sparsity of components and thus to a reduced regularization error. This is supported by the fact that the improvement is larger for reconstruction with L1-Norm compared to L2-Norm.

**Fig. 9:**
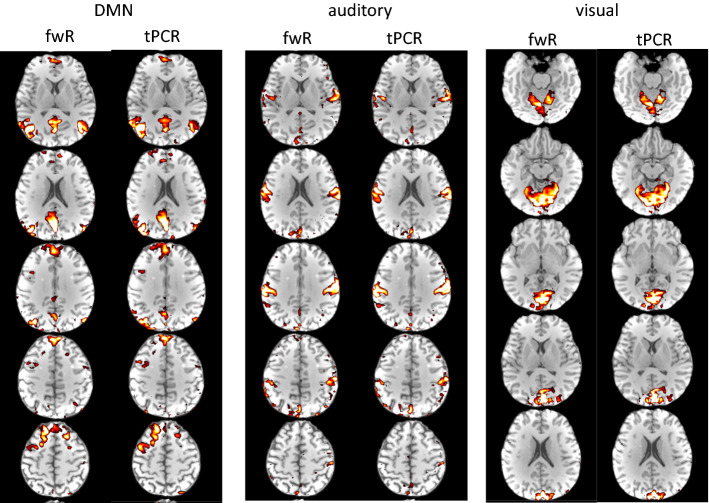
Comparison of ICA-based RSN-analysis for frame-wise reconstruction (fwR) with temporal principle component reconstruction (tPCR). Detected default mode network (DMN), auditory, and primary visual networks are virtually identical for both reconstruction modes

A breathhold paradigm was used for experimental demonstration in [48]. tPCR works equally well for task fMRI as well as resting state fMRI. Figure 9 demonstrates results from a resting state examination showing practically identical RSNs for frame wise reconstruction compared to tPCR, but with a reduction in overall computation time of a factor of ~ 5 for tPCR.

**Fig. 10:**
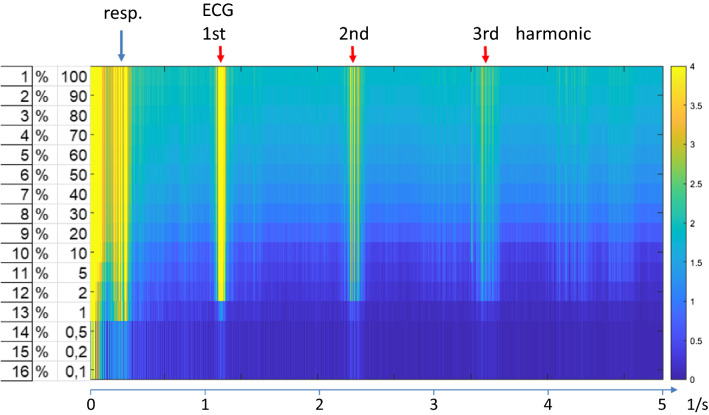
Image representation of frequency spectra of signal timecourse as a function of the percentage of PCA-components used in the final reconstruction. The horizontal axis represents frequency, the vertical axis represents the percentage of PCA-components used in the final recombination of the PCA-components. Signal intensities are scaled in arbitrary units, the yellow bands represent physiological signals as indicated at the top

**Fig. 11:**
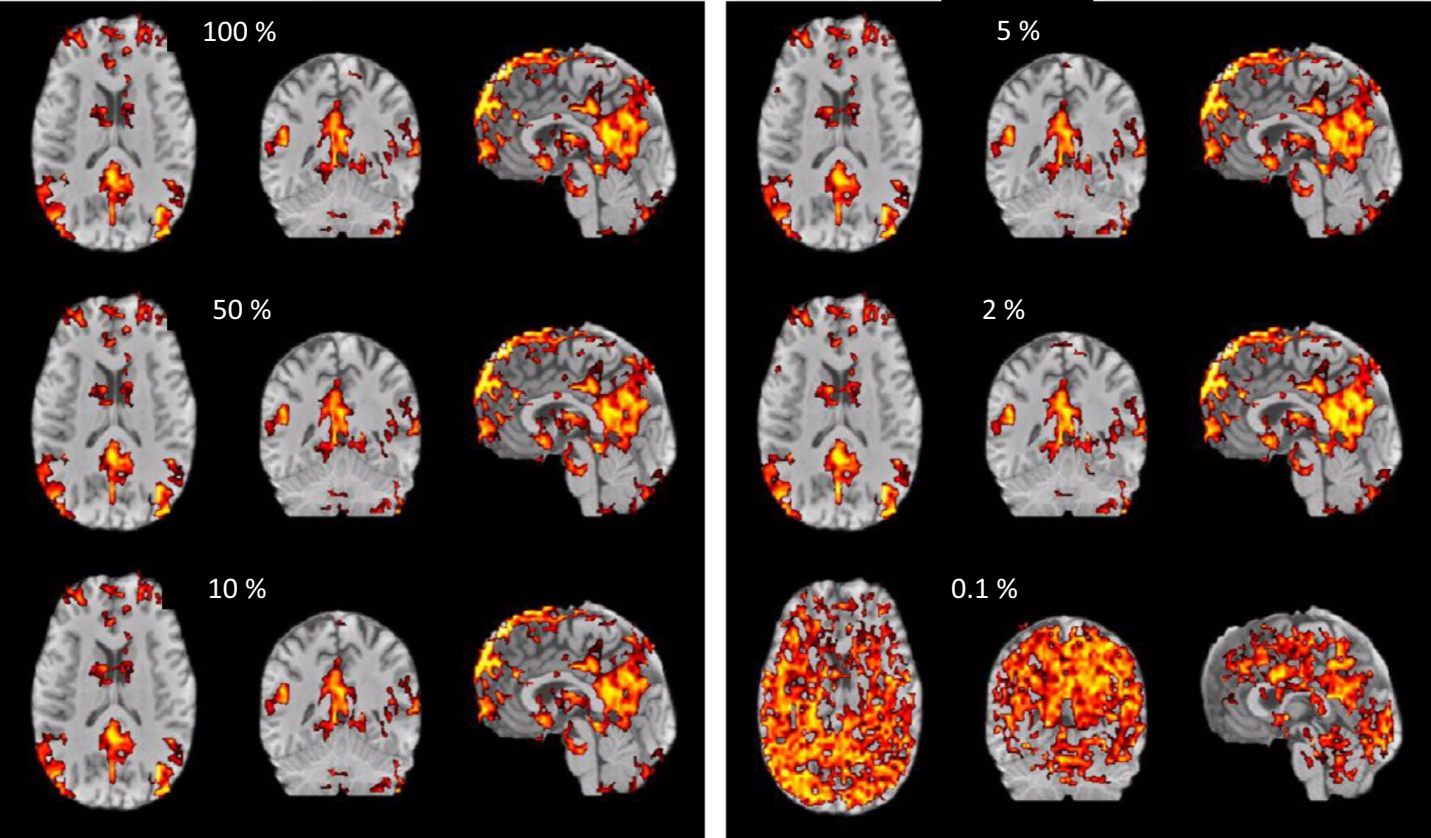
ICA reconstruction of the default mode network using a different percentage of the PCA components. In the example shown even 2% of the components deliver nearly identical networks compared to full reconstruction. Only at 0.1% network reconstruction breaks down

As a next step we are currently looking into how many PCA-components are actually necessary to reliably detect activation. Figure 10 shows as a preliminary result the global frequency spectrum as a function of the percentage of components used. The main effect of leaving out the higher order components is a reduction of the noise level in between the peaks of the physiological signals while the signals themselves stay (nearly) constant. Only towards the very low end when < 5% of the components are used the physiological signals suddenly vanish. Figure 11 demonstrates that this also applies for the detection of RSNs. ICA-analysis of a dataset with a varying percentage of components used shows hardly any change in the detected networks even when much < 10% of the components are used (movie in supplementary materials). A thorough analysis of the sensitivity to detect RSNs as well as in task based fMRI is currently under way. It should be noted that the gain in computation speed does not directly scale with the percentage reduction of components, since more iterations are required for the low order components (s.Fig. 2 in [48]). Still a reduction of computation time by another factor of 5 appears to be feasible.

### Activation studies

Having resolved many of the technical aspects associated with the fast image acquisition and reconstruction, there was great excitement to investigate how MREG could improve the results of practical fMRI applications. Following the analysis of simple fMRI paradigms such as visual or motor tasks, it became immediately obvious that MREG led to the detection of much stronger functional activations compared to EPI [15]. A broad range of fMRI applications could benefit from this improved sensitivity, ranging from research into subtle brain activity changes that could now be more easily detected, to clinical functional mapping scans that might potentially be considerably shortened without having to sacrifice statistical power.

**Fig. 12:**
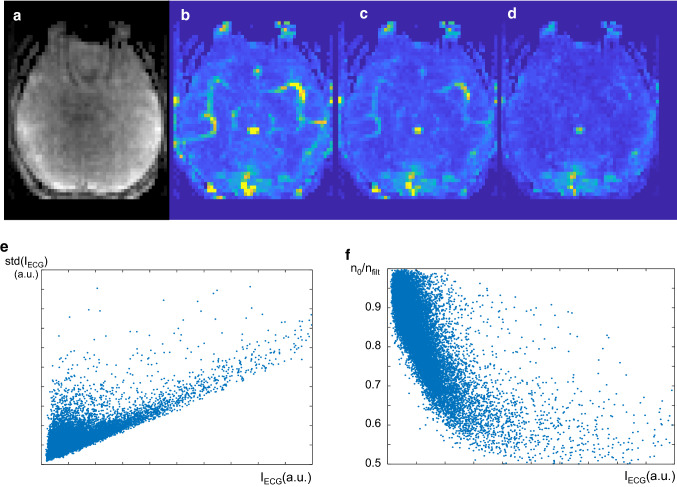
**a** Maximum intensity projection (MIP) through a 4.8 cam thick slab at the height of the visual cortex, (**b**) corresponding MIP of the pixel-wise temporal noise measured as the standard deviation of the signal timecourse after linear detrending. **c** MIP of the pixelwise temporal noise after filtering out the ECG-peak at 1.2 ± 0.1 Hz from the frequency spectrum. **d** MIP of the temporal noise after low pass filtering with a cutoff frequency of 1 Hz. **e** Plot of the signal intensity of the frequency peak at the ECG-frequency against temporal noise shows a clear correlation **e**. **f** Reduction of temporal noise after filtering out the ECG. Peak alone (corresponding to **c**)

Yet, it is actually not trivial why the high temporal resolutions achieved by MREG should yield higher fMRI sensitivities. While it had long been recognized that fMRI analyses would benefit from shortened TRs, this tendency had been expected to break down as TRs reached below ~ 1 s [49]. This is because TRs much lower than the T1 relaxation time (~ 1300 ms in gray matter at 3 T) only allow partial recovery of the longitudinal magnetization, necessitating the use of lower flip angles and yielding a lower MR signal amplitude (and corresponding lower SNR) which more or less balances out the improved sampling efficiency of 3D-acquisitions. However, statistical power in fMRI is only partially dependent on image SNR, since noise in fMRI time series is actually a combination of both thermal noise, on which SNR is based, and physiological noise, which depends on MR signal strength [50]. Analysis of the signal variation observed in MREG demonstrates that contributions from ECG-pulsatility contribute a large part of the physiological noise. Figure 12a shows the maximum intensity projection (MIP) through a 4.8 cm thick slab at the height of the visual cortex, and Fig. 12b shows the corresponding MIP of the pixel-wise temporal noise measured as the standard deviation of the signal timecourse after linear detrending. Although no vessels are seen in (a), vascular signals clearly stand out in (b) due to their high pulsatility. A plot of the signal intensity of the frequency peak at the ECG-frequency against temporal noise shows a clear correlation (e). Filtering out the ECG-peak at 1.2 ± 0.1 Hz from the frequency spectrum leads to pronounced reduction of vascular signals (c), which are, however, still clearly visible. A plot of the reduction of temporal noise after filtering (f) shows that this alone reduces temporal noise by up to 50%. In spite of the reduction, vessels are still clearly visible in (c). After low-pass filtering with a cut-off of 1 Hz most (but not all) vascular signals vanish.

**Fig. 13:**
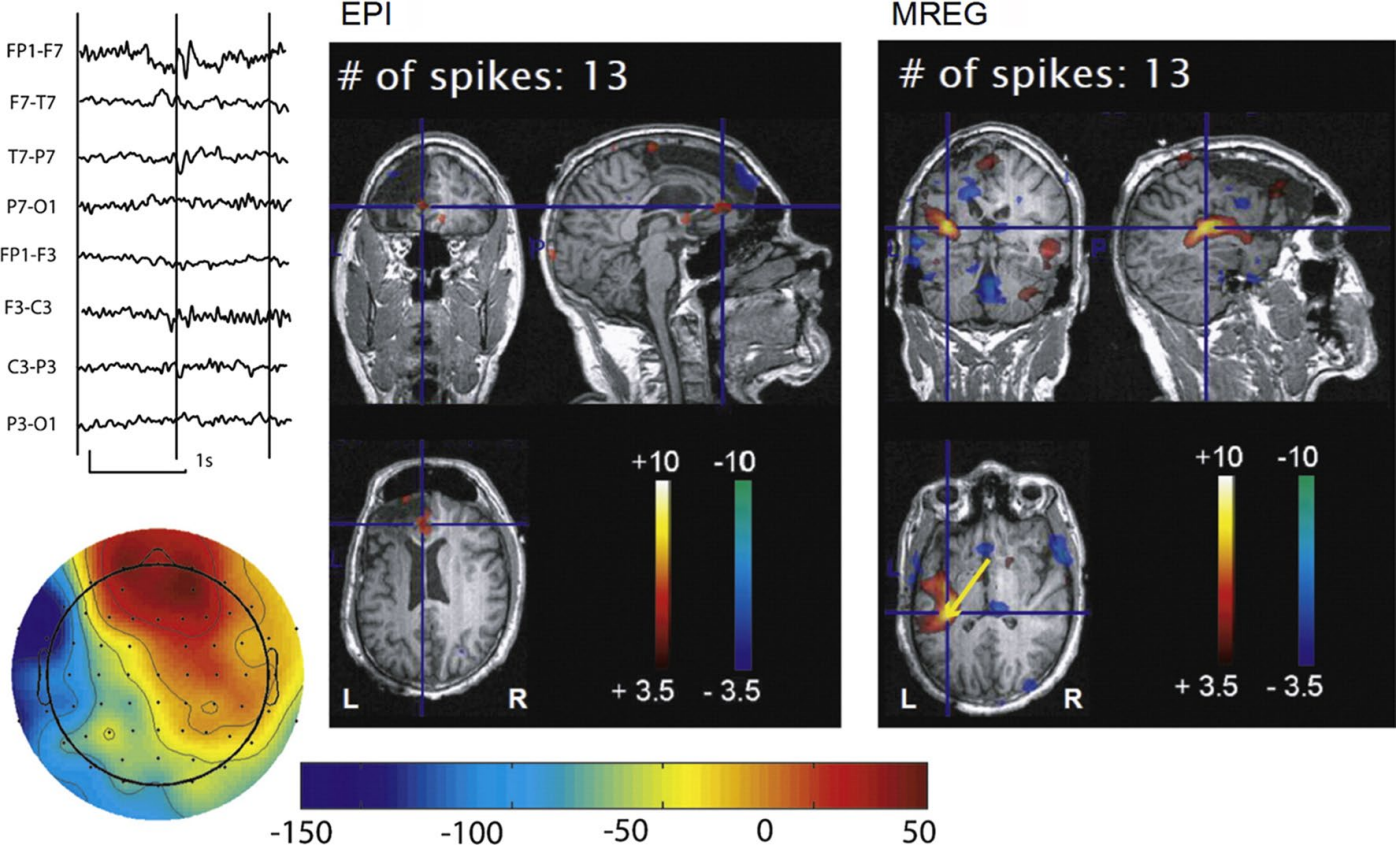
BOLD activation maps associated with left frontal epileptic spikes in a patient with focal cortical dysplasia with a previous frontal resection that did not result in seizure freedom. While the EPI map shows a small activation near the resection border, the MREG map reveals a much larger activated area extending into parietal regions. In the left upper corner a typical epileptic spikes is visible in the EEG trace over the left fronto-temporal area as well as a voltage map derived from this spike. (Fig. 5 from Jacobs et al. [52]) adapted from

This clearly demonstrates that at the typical spatial resolutions employed in MREG (3 mm isotropic), physiological noise dominates over thermal noise, so that even significant reductions in SNR will only result in small degradations in temporal SNR (tSNR) [51]. Indeed, we investigated BOLD activations associated with interictal epileptic discharges recorded with simultaneous EEG; such studies often show low sensitivity, yet are highly clinically relevant for the presurgical evaluation of focal epilepsy patients. Compared to conventional EPI acquisitions, MREG only resulted in a 18.4% reduction in tSNR, and this was more than compensated by the increased statistical power from the high temporal resolution, resulting in a much increased detectability of epileptic brain areas (Fig. 13; [52]).

As for why a higher temporal resolution yields higher fMRI sensitivity, this is primarily because the higher number of time points directly leads to higher noise degrees of freedom in the fMRI time series analysis, yielding more reliable estimates of statistical parameters. The same effect arises when increasing the length of the scan, but this is of course a more expensive alternative. A key point, however, is that a longer scan yields additional fMRI time points that are largely independent of the initially acquired time points, so that the increase in degrees of freedom matches the increase in the number of time points, i.e., a doubling of the scan length, for example, yields a doubling of the degrees of freedom. In contrast, additional time points resulting from a higher temporal resolution are not independent of each other. As BOLD time series primarily measure vascular signals that are physiologically sluggish, neighboring fMRI time points exhibit a high degree of correlation, especially at high temporal resolutions. As such, the increase in degrees of freedom is actually lower than the increase in the number of time points. The statistical analysis must then correctly model the time series autocorrelations and degrees of freedom to avoid any overestimate of the statistical parameters and corresponding loss of specificity. The groundwork for such modeling had been established early in the history of fMRI by Worsley and Friston [53]. Yet, the proposed models and subsequent implementations in fMRI statistical analysis packages only considered temporal autocorrelations in conventional, low temporal resolution fMRI data. Initial activation studies using fast fMRI data thus reported very large statistical scores that were, however, somewhat inflated [15, 28].We thus started to perform subsequent data analyses using the FMRISTAT toolbox, which is not widely used, but which allows modeling the noise as a spatially varying, high-order autoregressive process [54, 55]. This provided a well-fitting model of MREG time series autocorrelations while reducing the inflated statistical scores, which were nevertheless still ~ 60% higher than the scores obtained with conventional EPI [52]. An even higher detectability could be obtained using the ability of MREG to characterize the region- and patient-specific hemodynamic response function at high temporal resolutions [56]. This higher sensitivity notably allowed the detection of widespread cerebral areas associated with epileptic discharges [57–59]. However, there was still some evidence of bias in the statistical analysis [60]. It is only recently that analysis methods better suited to fast fMRI have emerged, which now allows to take full advantage of high temporal resolution data in fMRI activation studies [34, 61, 62].

### Resting-state networks

**Fig. 14:**
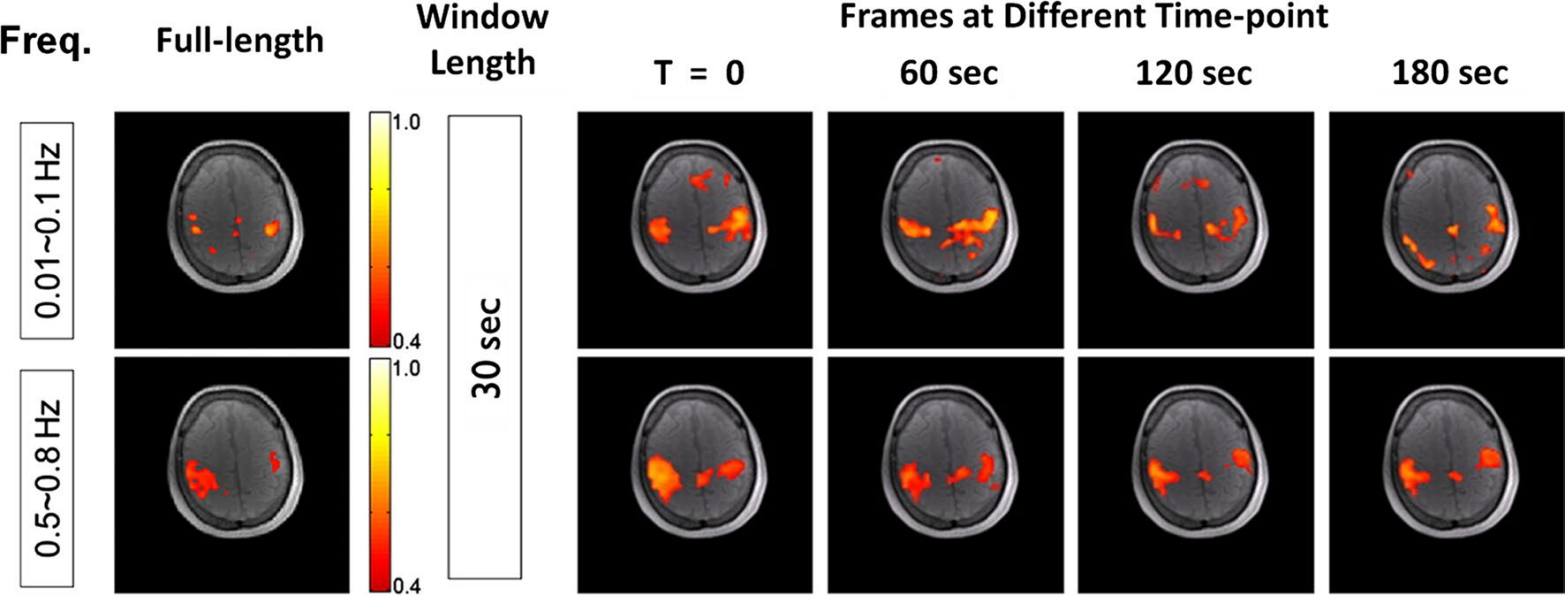
Resting-state connectivity in the primary motor cortex in the 0.01–0.1 Hz (top row) and 0.5–0.8 Hz (bottom row) frequency bands. While the connectivity calculated over the full-length scan (left column) involves the same motor areas in both frequency bands, the connectivity calculated over 30-s time windows (right columns) is highly variable in the 0.01–0.1 Hz band, also involving spurious regions outside motor areas. The calculated sliding-window connectivity is much more reliable in the 0.5–0.8 Hz band (adapted from Fig. 4 from Lee et al. [67])

**Fig. 15:**
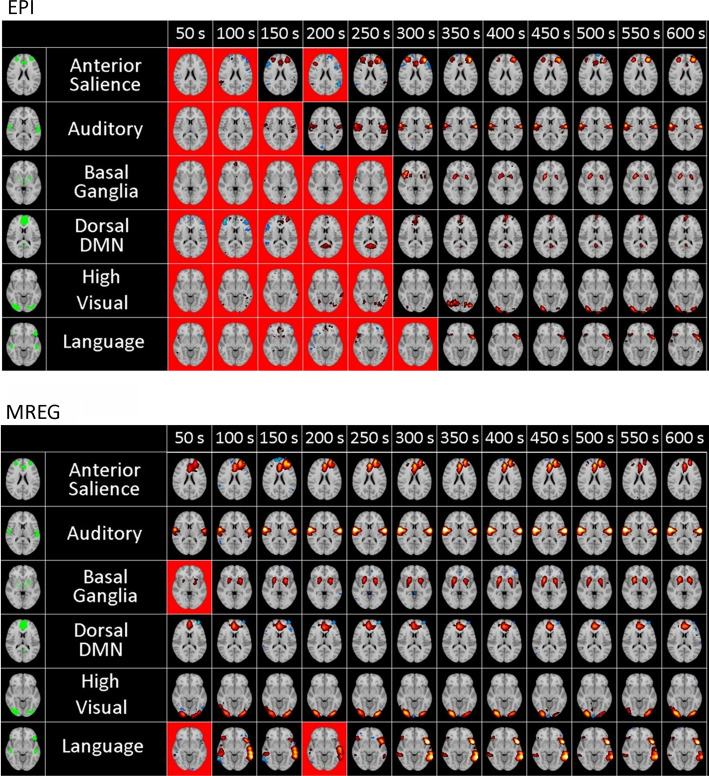
Common resting-state networks extracted by ICA from EPI (top) and MREG (bottom) data using various time window lengths (shown in the multiple columns). Images with a red background denote that a component corresponding to the given network could not be found. For time window longer than 300 s, all networks could be successfully detected with both sequences. However, for shorter time windows, the detection is only reliable when using MREG data. (adapted from Figs.  7, 8 from Akin et al. [69])

More than high statistical power, MREG opened up novel opportunities for the characterization of BOLD time series that had not been previously accessible at lower temporal resolutions. While fMRI signals have long been considered to be slowly varying in time, there was now the possibility to explore whether faster BOLD fluctuations could be physiologically meaningful. In addition to cardio-respiratory pulsations, which are no longer aliased at high temporal resolutions [63], high-frequency signals may also include BOLD-related temporal variations, for example due to fast transients in the hemodynamic response, which have long been described as potentially more direct reflections of neuronal activity than the slow hemodynamic peak [64–66]. Moreover, there is evidence that the hemodynamic response can actually rise and recover much faster than previously thought, particularly during the resting state, which would then manifest as BOLD fluctuations at higher frequencies. We thus investigated high-frequency functional connectivity within known resting-state networks [67]. Even when the MREG time series were bandpass-filtered between 0.5–0.8 Hz, thus completely removing the slow 0.01–0.1 Hz fluctuations typically associated with BOLD signals (and also avoiding frequency bands associated with respiratory and cardiac pulsations), strong connectivity was observed in primary visual and motor networks (Fig. 14). Moreover, the high-frequency networks were much more stable than the corresponding low-frequency networks. This was especially the case when considering connectivity patterns calculated over small time windows, as short as 30 s. This follows from the fact that a time window must be of sufficient length to cover at least one full cycle of a given oscillation, and therefore, slow oscillations require longer windows [68]. Further investigations using independent component analysis revealed that a more reliable extraction of functional connectivity patterns in short time windows could be obtained with MREG in all common resting-state networks (Fig. 15; [69]).

As resting-state networks could now be characterized in short time intervals, this may suggest that MREG could be used to dramatically shorten the length of resting-state fMRI scans without sacrificing sensitivity. However, functional connectivity is known to vary over time, even during a supposedly stable resting state. These dynamic connectivity fluctuations could be potentially relevant biomarkers of brain function and can only be captured over the course of several minutes [70]. High temporal resolution fMRI can nevertheless be highly beneficial, since it can reliably measure functional connectivity variations occurring over short time scales, even as these fast fluctuations occur as part of a longer scan overall [71, 72].

To facilitate the investigation of fast BOLD fluctuations, it was then natural to combine MREG with simultaneous EEG, whose high temporal resolution would provide useful baseline recordings of cerebral activity. While simultaneous EEG-fMRI had been previously almost exclusively used with EPI, it was quickly established that fast acquisitions and spiral trajectories did not cause additional safety issues [40, 73], nor prevent an effective removal of MR gradient artifacts from the EEG [74, 75]. As a starting point, we focused on the default-mode network (DMN), which is associated with the brain’s baseline state, giving it a central role as a hub of global brain activity [76]. Time-varying correlations between BOLD and EEG oscillations closely tracked dynamic DMN connectivity, supporting the neurophysiological origin of fMRI dynamic connectivity [77]. A case report in an experienced meditator who experienced a state of so-called “content-free awareness” further suggested that dynamic EEG and DMN connectivities were modulated by the state of consciousness [78]. We thus expect fast fMRI to keep playing an important role as the field of dynamic connectivity analyses continues to grow.

While resting-state functional connectivity is based on time series correlations, which are undirected measures, one may wonder whether it may be possible to infer the directionality of the connections from the fMRI data. In particular, high temporal resolution fMRI can allow the detection of small propagation delays of neuronal activity. This was well demonstrated by Fa-Hsuan Lin using inverse imaging, where time delays of hundreds of milliseconds in visual and motor areas could be clearly resolved at the group level [79]. In individual subjects, the spatial variability of the hemodynamic response function is, however, a major confound [80], but the results nonetheless showed that at the group level, inferences on hemodynamic delays could still be reliably performed [79]. Using MREG, we could thus characterize the dynamic lag structure of the DMN [81, 82].

Yet, there was still interest in inferring directed connections from fast fMRI data independently of hemodynamic delays, which would allow the analysis to be performed at the individual level. We thus developed a method to estimate the directed effective connectivity from the undirected covariance matrix of the BOLD time series [83]. The method is based on the fact that so-called “collider” structures, in which two uncorrelated variables contribute to the activity of a third variable, lead to specific entries in the inverse covariance matrix. With the aid of a sparsity prior, it then becomes possible to identify a unique directed network structure that best explains the observed data. We could additionally show that the estimation was much less sensitive to hemodynamic variability than lag-based methods, and that the connectivities were more consistent when calculated using high-frequency than low-frequency BOLD fluctuations [84]. The measurement of these high-frequency temporal signals crucially depended on the high temporal resolution provided by MREG.

### MREG-based imaging of physiological brain pulsations

Hans Berger, during his development of electroencephalography in early 1900s, described three basic brain waves within the brain: “eine pulsatonische, eine respiratorische und vasomotorische Bewegung” [85]. In 1995 Bharat Biswal connected the vasomotor waves as a phenomenon related to the spontaneous functional connectivity fluctuations in primary sensory cortices [86, 87]. Over decades, the two other physiological brain pulsations, i.e., the cardiorespiratory pulsations, have largely been regarded as noise that obscures and aliases over cued and spontaneous brain activations in the fMRI BOLD signal [88–90].

However, the physiological noise structure in even relatively slowly sampled (TR > 1 s) fMRI BOLD signal has gained increasing interest in both physiological and clinical viewpoints. Heart rate variability and respiration has been shown to be modulating cognitive performance and brain activation responses [91, 92]. Standard deviation as well as variance of the BOLD signal itself has been shown to correlate significantly with pathological conditions like Alzheimer’s disease [93–96], small vessel disease [97], stroke [98], and chronic kidney disease-related changes in brain [99].

The interest in brain pulsations as a source of valuable new information have surged after the discovery of the glymphatic brain clearance system in 2013 by Nedergaard [100]. The glymphatic mechanisms have been shown to be a key element in several major forms of neuropathology including Alzheimer’s disease, stroke, trauma, and epilepsy [101–109]. The glymphatic brain clearance is driven by vascular pulsations that convect both brain metabolites and waste along CSF water in paravascular spaces in humans and mice [110, 111].

Glymphatic brain research has mostly focused on following contrast media flow along periarterial CSF spaces in mice in vivo microscopy and MRI techniques [100, 110, 112]. In humans the contrast media has been injected in lumbar intrathecal space or directly into brain interstitium via capillary blood brain barrier-opening in therapeutic AD and ALS studies [113–115]. The human interstitial brain tissue has been shown to become cleared from contrast media in clearly supra-diffusive speeds by the glymphatic mechanism [113–115].

Although the contrast media studies precisely map the epitope paravascular convection of foreign materials in the glymphatic system of the brain, the invasive nature of these studies makes them less feasible for routine large scale clinical settings. Instead of invasive MR-contrast media injections, different novel approaches in estimating brain water molecule transport have emerged and these could give information on how different diseases affect the glymphatic mechanism [116–118].

### Ultrafast scanning as a tool for physiological pulse mapping.

**Fig. 16:**
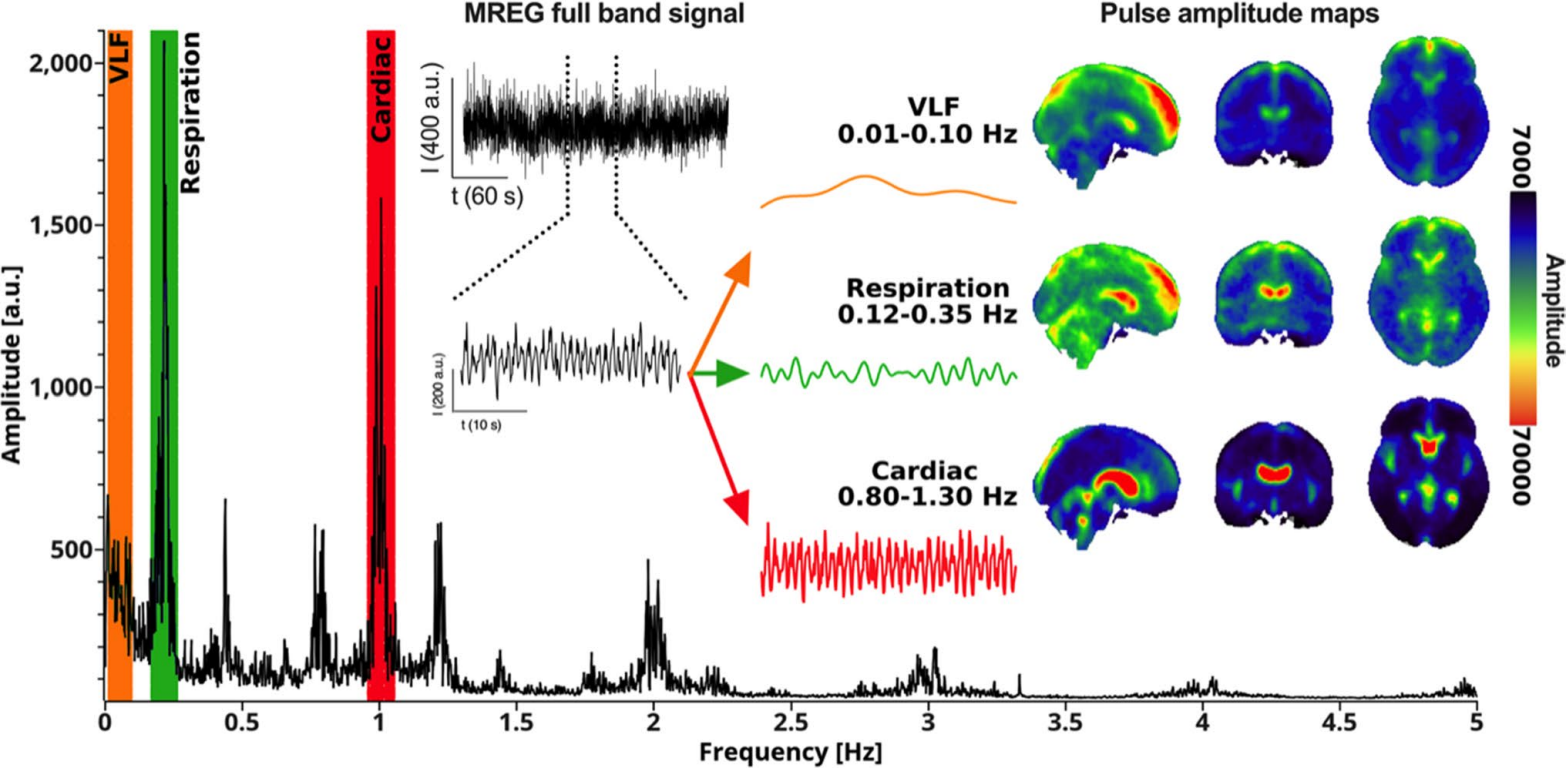
Example of human full band MREG signal with FFT power spectrum presenting three main physiological pulsations in the spectrum peaks. MREG signal was further band passed for quantification and mapping into anatomy. The VLF band and representative signal in orange, respiratory pulsations in green and cardiac pulsations in red, respectively, overlaid over MNI 152 space. Please also notice the harmonic power spectrum peaks over the full 5 Hz power spectrum highlighting the precision of the MREG signal

With the emergence of ultrafast scanning sequences like MREG it now became possible to detect physiological brain pulsations over the whole brain with critical sampling rates < 300 ms thatavoid pulse aliasing [72, 119]. As can be seen from the MREG BOLD signal in Fig. 16, both the cardiac and respiratory present clear brain pulsations that can be separated from the very low frequency (VLF < 0.1 Hz) pulsations and also from 1/*f *thermal noise. The fast scanning offers a clear measurement of the pulsations that may offer an alternative and more accurate physiological image contrast, along with the previously stated increases in statistical power.

In microscopical in vivo imaging the main drive of the glymphatic convection has been related to cardiovascular pulsations. In humans; however, the known three sources of brain pulsation introduce a more macroscopical scale effect that can also be detected by fast MRI scanning [119–121]. The cardiovascular pulsation induces a centrifugal pulse in the brain directed from the center outward in sync with the cardiac cycle, and the respiration induces a more centripetal pulse extending from the brain surface towards the center. Both of these pulses also affect the CSF spaces throughout the brain, most remarkably in the ventricles [119]. The very low frequency (VLF) vasomotor waves then present slowly moving patterns of seen over brain cortex that can also be seen in conventional fMRI BOLD signals [72, 122–124].

It seems that the source of the detected MREG signal changes in cardiorespiratory frequencies originate from vascular as well as extravascular sources like the water movement impulses that the physiological pulses induce inside the brain [125]. The BOLD signal is reflecting spin coherence from intra and perivascular water protons as they are being modulated by regional deoxyhemoglobin (Hb) concentration (2/3) and blood volume (1/3) as a slow response to neuronal activity [126]. In contrast to previous beliefs during the birth of theories on BOLD signal origins, the glymphatic research clearly shows that the water (and the molecules/aggregates it carries) does not stand still in paravascular space but is driven by physiological pulsations.

The cardiovascular impulses introduce propagating waves of spin coherence perturbations that traverse the very liquiform brain tissue which are captured by the MREG signal. Both echo-volumar imaging (EVI) as well as MREG have detected a drop in arterial signal intensity upon arterial impulse arrival and this impulse amplitude reduces when moving along the tissue [72, 119, 127]. This signal change in the (peri)arterial areas is dominated by the cardiac impulse by flow effects. As the arteries continuously have > 97% SpO_2_ saturation, the BOLD effect from Hb is minimal.

Towards the capillaries and especially in the veins/venules, the [Hb] concentration increases and BOLD effect starts to dominate. Similarly the cardiac flow impulses become weakened into a more laminar steady state flow that enables better gas exchange between tissue compartments.

The respiration related pressure changes in the thorax are the main driver of both the intracranial CSF flow and venous blood return. The incompressible skull along with the only incompressible venous sinuses in the body surrounded by dura mater around the brain and spinal cord introduce unique pressure conductance environment for the CNS venous sinuses. This unique physiological construction makes it possible that the pressure changes in the thoracic area are readily conducted into the brain and spinal veins [108].

While the inspiration draws venous blood from the brain, the cerebrospinal fluid produces a counter inflow as the rigid cranial volume stays constant [120, 121]. The same mechanism may take place in the penetrating paravenous space and cortical veins creating efflux waves leading waste material out of the CNS. The respiratory pulsation affects both the venous blood oxygenation and volume and thus affect T2* weighted BOLD signal in MREG measurements; the route is reverse to normal activation hyperemia but is based on known venous blood flow physiology and it has now become more accurately accessible via fast scanning sequences.

### Clinical relevance of physiological pulsations

**Fig. 17:**
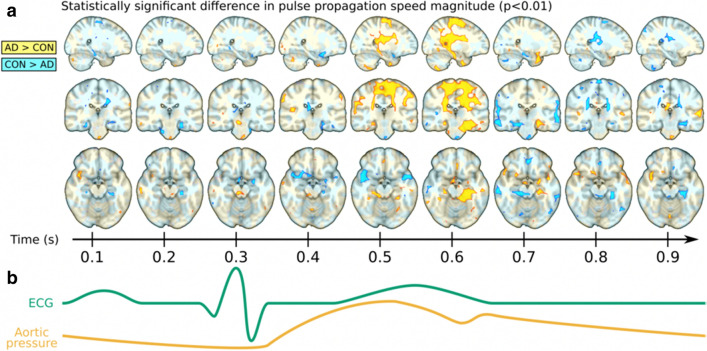
Example of optical flow analysis of MREG cardiac pulse propagation over the brain from the Alzheimer brain analysis revealing momentary pulsation abnormality occurring during the cardiac impulse arrival in the brain. The abnormality is highly variable over time and space and affects the BOLD signal variance significantly, see also [81]. Due to critical 10 Hz sampling rate of the MREG, the abnormality can be quantified with unprecedented spatiotemporal precision. To see how the cardiac impulse propagates over the brain in video, please see also: https://www.newscientist.com/article/mg23130864-200-best-look-yet-at-how-our-brains-sewage-system-flushes-out-waste/

Early MREG experiments have given encouraging results with respect to clinically feasible diagnostic markers. In line with previous analyses of BOLD signal variance, we have been able to show with three distinct datasets that the brain BOLD signal variance is altered in Alzheimer’s disease [93, 94, 96, 97, 99]. An example is shown in Fig. 17.

Furthermore, the source of the abnormal variance seems to be the cardiovascular rather than any other pulsation source. A novel approach in analyzing the physiological brain impulse propagation in the brain—based on optical flow analysis—was able to detect markedly altered variance also in the propagation of cardiovascular brain impulse in Alzheimer brains [128]. Further analyses indicate more pronounced abnormalities in the directionality and magnitude of brain pulsations as well (Rajna et al., submitted).

Intractable epilepsy has been shown to be related to AQP4 water channel absence from the perivascular astrocytic endfeet lining the perivascular glymphatic space [129]. The AQP4 molecule is a key molecule for the glymphatic clearance of the interstitial space as the removal of b-amyloid in AQP−/− knockout mice is reduced by 60% [130]. The brain signal variance was shown to be altered in intractable epilepsy patients in the respiratory frequency range [131]. Intracranial EcoG needle measurements in intractable epilepsy patients have also demonstrated a strong drive of brain activity LPF, MUA by respiratory brain pulsations [132].

**Fig. 18:**
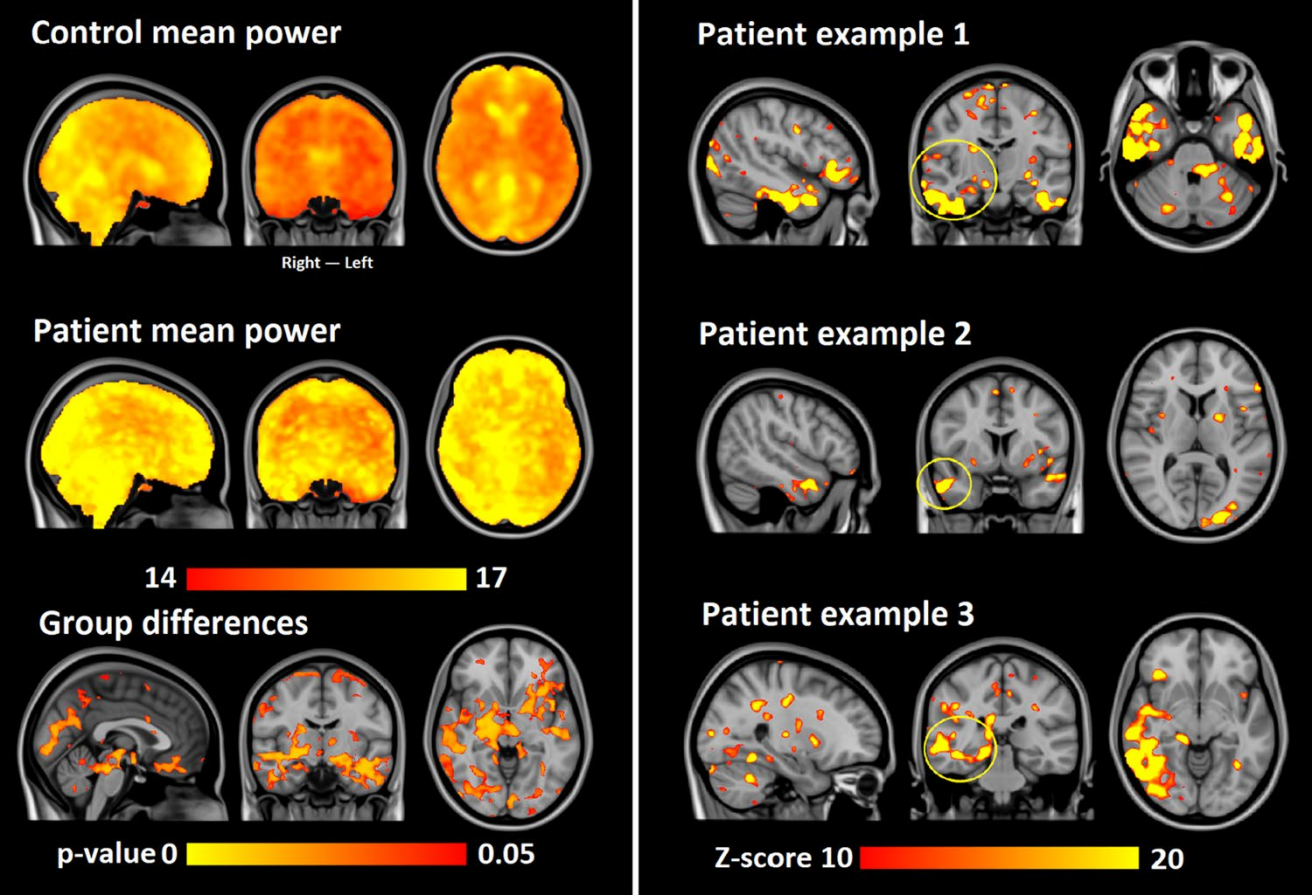
The respiratory pulsation power is altered significantly in epilepsy. Top: the mean of control and patient respiratory brain pulsation power. Bottom: the significant pulsation power changes (*p* < 0.05 FSL randomize TFCE-corrected for voxel-level). Right panel: patient examples showing individual patient’s increase > 10 standard deviations above control (*n* = 100) respiratory pulsation power

Interestingly the MREG data was able to show individual abnormalities of brain signal variance in two consecutive scans compared to age-matched control population. We have enlarged the MREG scan population into a two-center study and detected a repeated abnormality of epileptic brain pulsatility abnormality > 6 standard deviations above normal mean values (Kananen et al., submitted) (Fig. 18). This in practice means that individual-level changes can be seen in patients, which has not been possible with prior BOLD scanning methods. The critical sampling rate of the MREG signal ensured separation of pulsations and offers needed statistical power that enables individual level diagnostic capability. Furthermore, spectral entropy of the MREG and other multimodal data (EEG, NIRS) obtained simultaneously suggest a spectral entropy alteration in intractable epilepsy [133].

## Discussion and outlook

The concept of MREG originally formulated as a mere ‘Gedankenexperiment’ has come a long way since its original conception. Originally it was meant to produce time series signals from rather large volumes selected by coil sensitivity profiles alone, but it quickly evolved to produce quite decent looking 3D-datasets in a time, that is still short compared to ‘proper’ imaging. It has to be said that with the parallel development of compressed sensing the boundary between the concepts of MREG and ‘proper imaging’ have been smeared out, indeed MREG can be regarded as a rather extreme implementation of compressed sensing with a high total undersampling factor of 18 for a 3D-acquisition. There is no magic associated with the ultrafast sampling speed of MREG. The key factor is to use a trajectory which allows isotropic undersampling in all three spatial dimensions. The undersampling factor of 2.6 in each direction is still within the theoretical limits demonstrated by Wiesinger [134]. Other trajectories offer similar acceleration, amongst the three types we have used (rosettes, concentric shells, stack-of-spirals) the latter offers the most benign artifact behavior. One lesson we learned in going through various types of trajectories is that intersections (like rosettes) are problematic and may easily lead to artifacts.

With rectilinear trajectories one gradient (normally called the readout gradient) is almost by definition the ‘lazy’ one contributing little to acceleration, which has to be made up in the other dimensions.

Simultaneous multi-slice (SMS)-InI has reported a short TR of 100 ms but at the cost of even lower spatial resolution of 5 mm compared to MREG [135].

**Fig. 19:**
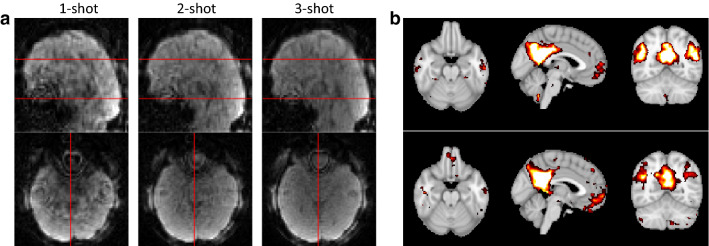
**a** Result from 1-shot, 2-shot, and 3-shot segmented MREG acquired with TR of 96, 180, and 264 ms, respectively, shows improvement in image quality of segmented acquisition. **b** Comparison of seed-based RSN for 1-shot (top) and 3-shot (bottom) trajectories shows nearly identical results

**Fig. 20:**
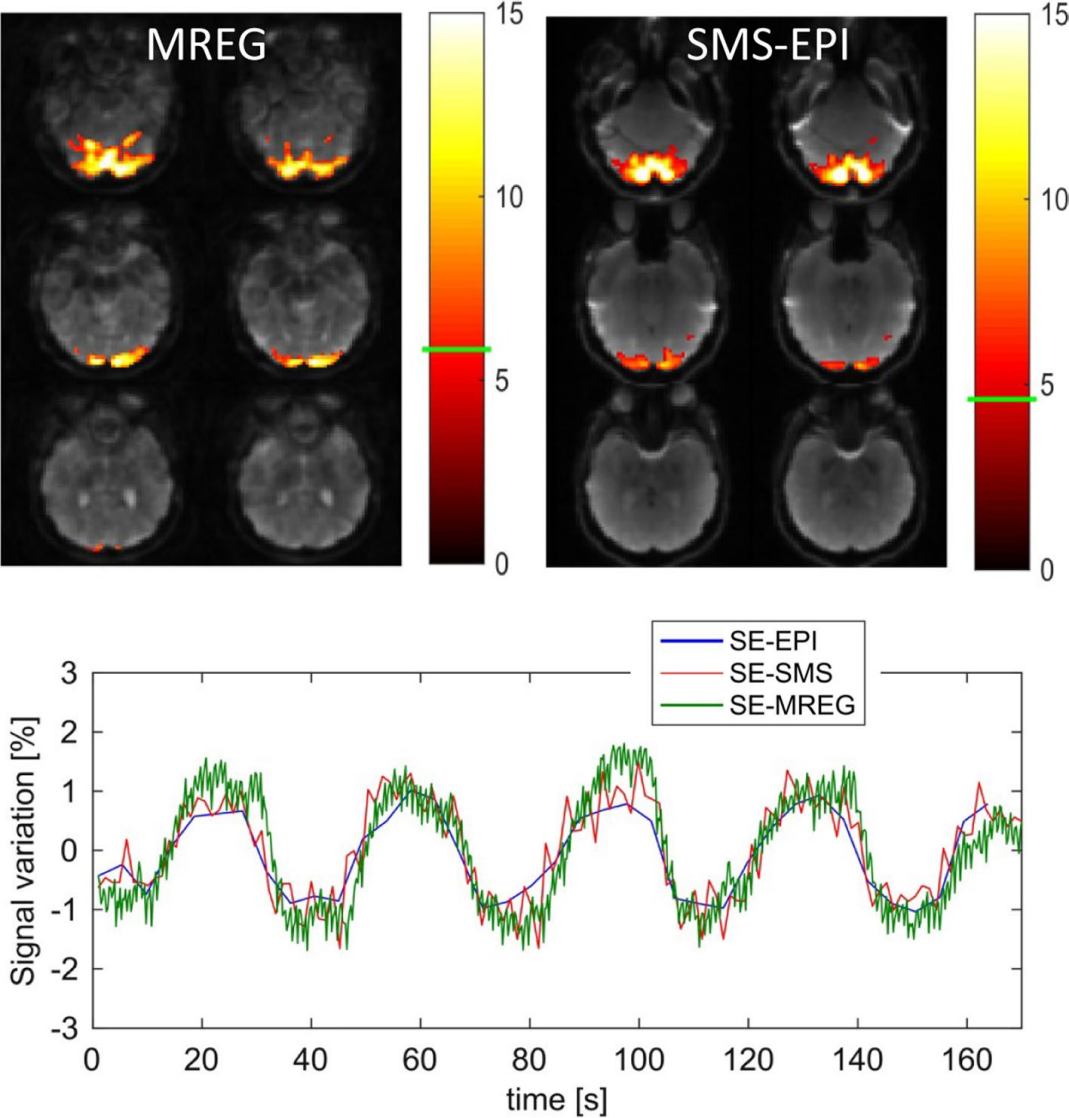
Comparison of activation maps acquired with spin-echo MREG (TR = 250 ms) (top left) and SMS-EPI (TR = 1300 ms) (top right) for checkerboard stimulation. Activation maps are overlaid to a single time frame of the measurement series. Both datasets had identical spatial resolution (3 × 3 × 3 mm^3^), stimulus was presented in a block paradigm with interval times 18 s on–18 s off. Color bars represent *t* values with the *t* threshold indicated by the horizontal green bar. The *t* threshold has been determined by the method of surrogate data [139] to account for the different number of data points as well as the different noise properties dependent on the temporal resolution. The bottom graph shows the signal time course in the activated areas, it also includes result from a standard EPI experiment

SMS-EPI has quite dramatically improved acquisition speed in conventional fMRI. Multiband excitation in combination with blipped z-gradients allows unprecedented acceleration in the *z*-direction. The rectilinear k-space trajectory yields good image quality and benign artifacts but allows only modest in-plane acceleration. The lowest reasonable TR before through-slice artifacts start creeping in has shown to be in the range of 400–600 ms [34] at a spatial resolution of 2.5 × 2.5 mm (in plane) and 3 mm slice thickness. It has indeed been a quite tantalizing problem to find a compromise between the high temporal resolution of MREG and the better image quality and spatial resolution of SMS-EPI. A combination of a stack-of-spiral trajectory with multiband excitation [136] has shown to bring some at least nominal improvement in spatial resolution, further improvements have been shown with a rotated stack-of-spirals partial acquisition by the same group of authors [137]. We have pursued a segmented approach that shows some improvement in image quality at longer TR of ~ 300 ms at still moderate spatial resolution of 3 × 3 × 3 mm^3^ (Fig. 19). As an alternate approach to improve image quality and to introduce spin-echo contrast we have implemented a spin-echo based MREG. This allows maintaining a still short TR of ~ 250 ms at the cost of lower SNR [138] (Fig. 20). We are still working towards our next goal which would be ~ 2 mm isotropic resolution at < 200 ms repetition time. We suspect that this will require not only methodological improvements, but also improved hardware including coil arrays with more elements as well as faster gradients. At the current status MREG is the method of choice for applications, where acquisition speed is the primary objective, if one can live with lower imaging speed, SMS-EPI wins out by its superior image quality. For higher fields like 7 T or beyond, rectilinear trajectories seem to become a necessity.

It would be actually quite tempting to try out MREG on low field systems, where it may offer decent anatomical imaging at unprecedented imaging speed. This would require a low field magnet with still high gradient performance—a not too common combination.

So far we have distributed the method to ~ 20 research institutes worldwide. The main obstacle to a more widespread distribution most probably lies in the long reconstruction times and necessity to perform reconstruction on a separate hardware. The improvement in reconstruction by tPCR together with new concepts for system architecture may change this in the not too distant future.
